# Management of prolonged first stage of labour in a low-resource setting: lessons learnt from rural Malawi

**DOI:** 10.1186/s12884-021-03856-9

**Published:** 2021-05-22

**Authors:** Wouter Bakker, Elisabeth van Dorp, Misheck Kazembe, Alfred Nkotola, Jos van Roosmalen, Thomas van den Akker

**Affiliations:** 1 Clinical and Nursing Department, St. Luke’s Hospital, Malosa, Malawi; 2 Athena Institute, Faculty of Science, VU University Amsterdam, Amsterdam, The Netherlands; 3 Department of Obstetrics and Gynecology, Leiden University Medical Centre, Leiden, The Netherlands

**Keywords:** Cephalopelvic disproportion, Caesarean section, Augmentation of labour, Amniotomy, Prolonged labour

## Abstract

**Background:**

Caesarean sections without medical indication cause substantial maternal and perinatal ill-health, particularly in low-income countries where surgery is often less safe. In presence of adequate labour monitoring and by appropriate use of evidence-based interventions for prolonged first stage of labour, unnecessary caesarean sections can be avoided. We aim to describe the incidence of prolonged first stage of labour and the use of amniotomy and augmentation with oxytocin in a low-resource setting in Malawi.

**Methods:**

Retrospective analysis of medical records and partographs of all women who gave birth in 2015 and 2016 in a rural mission hospital in Malawi. Primary outcomes were incidence of prolonged first stage of labour based on partograph tracings, caesarean section indications and utilization of amniotomy and oxytocin augmentation.

**Results:**

Out of 3246 women who gave birth in the study period, 178 (5.2%) crossed the action line in the first stage of labour, of whom 21 (11.8%) received oxytocin to augment labour. In total, 645 women gave birth by caesarean section, of whom 241 (37.4%) with an indication ‘prolonged first stage of labour’. Only 113 (46.9%) of them crossed the action line and in 71/241 (29.5%) membranes were still intact at the start of caesarean section. Excluding the 60 women with prior caesarean sections, 14/181 (7.7%) received oxytocin prior to caesarean section for augmentation of labour.

**Conclusion:**

The diagnosis prolonged first stage of labour was often made without being evident from labour tracings and two basic obstetric interventions to prevent caesarean section, amniotomy and labour augmentation with oxytocin, were underused.

**Supplementary Information:**

The online version contains supplementary material available at 10.1186/s12884-021-03856-9.

## Background

Globally, there has been a substantial increase in births by caesarean section, as described in the latest Lancet series on optimizing caesarean section use [1–3]. While this increase is most alarming in regions such as Latin-America and the Middle-East, there was also an increase in sub-Saharan Africa [1, 4]. This increase at population level is seen by some as a welcome development, since it may indicate improved access to caesarean section in rural areas where women were previously unable to access comprehensive emergency obstetric care [5, 6].

Ample evidence, however, exists that at facility level in sub-Saharan Africa, there is overuse of caesarean sections rather than underuse [7–12]. Many caesarean sections are performed without appropriate medical indication, suggesting that the observed increase at population level is not only a sign of improved access, but also of resorting to ‘too much too soon’ to reiterate the language applied in the Lancet series [1, 13]. The authors suggest that overuse of caesarean sections may result from lack of clinical experience and skills among maternity caregivers and fear of litigation [3].

Prolonged first stage of labour is one of the most frequent indications for caesarean section [14, 15]. This diagnosis is often based on presumed cephalopelvic disproportion (CPD) [16–18]. Since CPD is a diagnosis of exclusion and can generally only be made in presence of adequate uterine contractions, clinical decisions must be based on labour progress recording, for which the partograph is the commonly applied tool [19, 20]. When labour progress deviates significantly from the alert line or when the action line in the partograph is crossed, artificial rupture of membranes, also called amniotomy, and augmentation of labour with oxytocin are the recommended interventions to manage prolonged labour [14, 21, 22]. A relative contra-indication for oxytocin use is a previous caesarean scar, which necessitates frequent monitoring and awareness of the risk of uterine scar rupture [23–25].

In this study, we aimed to identify the incidence of prolonged first stage of labour and the proportion of prolonged first stage of labour as an indication for caesarean section in a rural hospital in Malawi, a country with a maternal mortality ratio of 634/100,000 live births and a neonatal mortality rate of 23/1000, belonging to the highest in the world [26, 27]. In addition, our objective was to assess use of artificial rupture of membranes and oxytocin augmentation to manage prolonged first stage of labour. This may identify opportunities to reduce unnecessary caesarean sections in similar settings.

## Methods

### Study design and setting

This retrospective study was performed from January 1st, 2015 to December 31st, 2016, in St Luke’s Hospital, a rural hospital in Malosa, Malawi. The 150-bed mission hospital provides free Basic and Comprehensive Emergency Obstetric Care for its catchment area. The maternity department consists of a labour ward with five birthing beds and an antenatal and postnatal ward. Antenatal care is provided in the out-patient department, following the former four-visit focused antenatal care model by the World Health Organization (WHO) [22]. A maternity waiting home is available for women at term who live far from hospital and are not in need of admission [28, 29]. Two health centres in the catchment area could refer ante- or intrapartum women with complications to St. Luke’s hospital. The hospital was equipped for births above an estimate of 32 weeks of gestation, since only relatively basic neonatal care was available. All women, however, who presented in labour or as referral were attended to and onward referral was only done if considered safe. Closest referral hospital was Zomba Central Hospital at 30 min by ambulance, one of four central hospitals in the country with a neonatal intensive care unit. In St. Luke’s Hospital, as in all hospitals in Malawi, births are attended by qualified nurse-midwives and student nurses, under responsibility of medical doctors or associate clinicians (clinical officers). In the study period, all shifts were covered by sufficient staff, however, day to day variation in staffing levels sometimes occurred.

### Interventions in labour

Amniotomy is a low-risk procedure that can be performed to expedite labour. It is preferably performed using a specially designed amniotomy hook, but sometimes other available instruments like Kocher’s artery forceps are also used. Caution is needed with umbilical cord presentation or fetal malposition. National and local protocols describe augmentation of labour with oxytocin as an additional option to expedite labour if deemed necessary. Oxytocin is diluted in normal saline, whereby the drop rate can be titrated in order to achieve regular contractions [30].

### Data collection

**Fig. 1:**
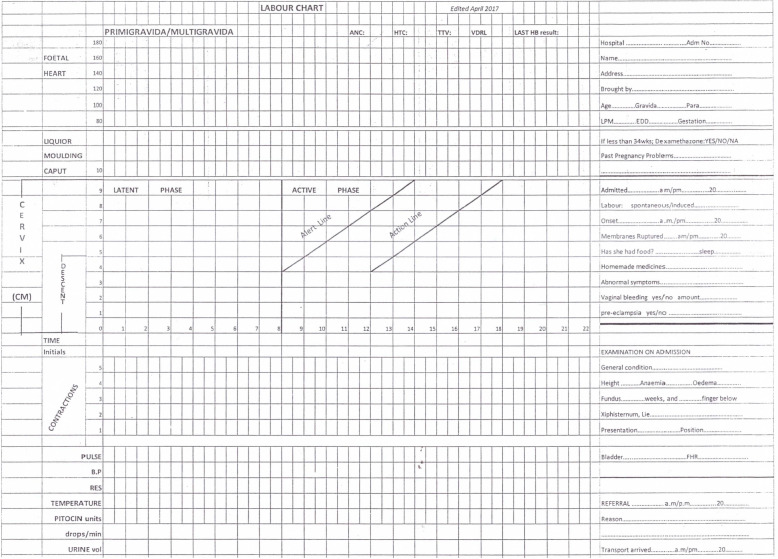
The labour chart with partograph used in St Luke’s Hospital, Malawi, adapted from the modified WHO partograph [20]. This labour chart is used for all women admitted to labour ward and contains all information on history, received antenatal care and progress of labour

All medical records of women who gave birth in the study period were collected and manually assessed by EvD, under supervision of WB. Characteristics of women as well as details of labour progress were retrieved from partographs and admission forms. In Malawi, a labour chart incorporating the modified WHO partograph is used (Fig. 1) [31, 32]. The partograph is a crucial document in maternity care, containing relevant information about risk factors, labour progress, outcomes and puerperium. Therefore, files without a partograph or with clearly incomplete partographs were not included. The partograph used in Malawi has an action line that progresses at one centimetre per hour and is plotted four hours (four boxes) to the right of the alert line (see Fig. 1). Cervical dilatation found during labour assessments is plotted on the partograph. If progress in cervical dilatation crosses the action line, it is recommended that an intervention to expedite birth is taken. This intervention could be amniotomy, augmentation with oxytocin or if those measures are already taken, caesarean section. We defined prolonged first stage of labour as crossing the action line of the partograph in the first stage of labour, which is after the latent phase and before reaching full dilatation of the cervix. The latent phase is considered the phase prior to active labour, generally thought to occur before four centimetre of dilatation, as indicated in the partograph. In the latent phase, which varies considerably in duration between women, contractions are irregular and labour progress is very slow and not yet plotted. Since the focus of this study is on prolonged first stage of labour, women with a clearly prolonged second stage of labour were excluded. Women who arrived in the hospital in the second stage of labour were not considered to also have a prolonged first stage of labour, unless these women had been referred from a primary health care facility and had a clearly documented partograph with prolonged first stage of labour.

All information was collected in an anonymized database containing 69 variables with information on maternal and fetal characteristics, history of current and past pregnancies, mode of birth and interventions during labour. The variables are listed in Additional file 1.

### Data analysis and outcomes

Data were analysed using SPSS, version 24. Women with prolonged first stage of labour were identified and compared with women with an uncomplicated progress of the first stage of labour. Both primi- and multiparous women who had caesarean section for prolonged first stage of labour were selected and assessed for rupture of membranes (spontaneous or artificially) and oxytocin use. All women who gave birth by caesarean section were identified and analysed, whereby indications where listed and use of interventions prior to caesarean was assessed. Apgar scores of neonates from women with and without oxytocin use were compared with a chi-square test.

## Results

In total, records of 3426 women were collected, of whom 2712 had spontaneous vaginal births, 69 (2.1%) assisted vaginal births and 645 (18.8%) caesarean sections. Most women attended the hospital as first point of care, while 197 (5.8%) were referred from other facilities. The majority of women (2724, 79.5%) arrived in hospital during the first stage of labour, while 104 women arrived during the latent phase. A considerable number of women arrived in the second stage (566, 16.5%), of whom 39 (6.9%) were referred from other health facilities. Fifty-two women only had a documented prolonged second stage of labour and were left out of further analysis, along with 135 (5.5%) women for whom it was unclear whether the action line was crossed (in total 187). Of these women left out of further analysis, a large proportion gave birth by mostly elective caesarean section (two previous caesareans, prelabour rupture of membranes in an HIV-positive woman) or for pre-labour emergency indications (cord prolapse, eclampsia, placental abruption).

### Prolonged first stage of labour

**Fig. 2:**
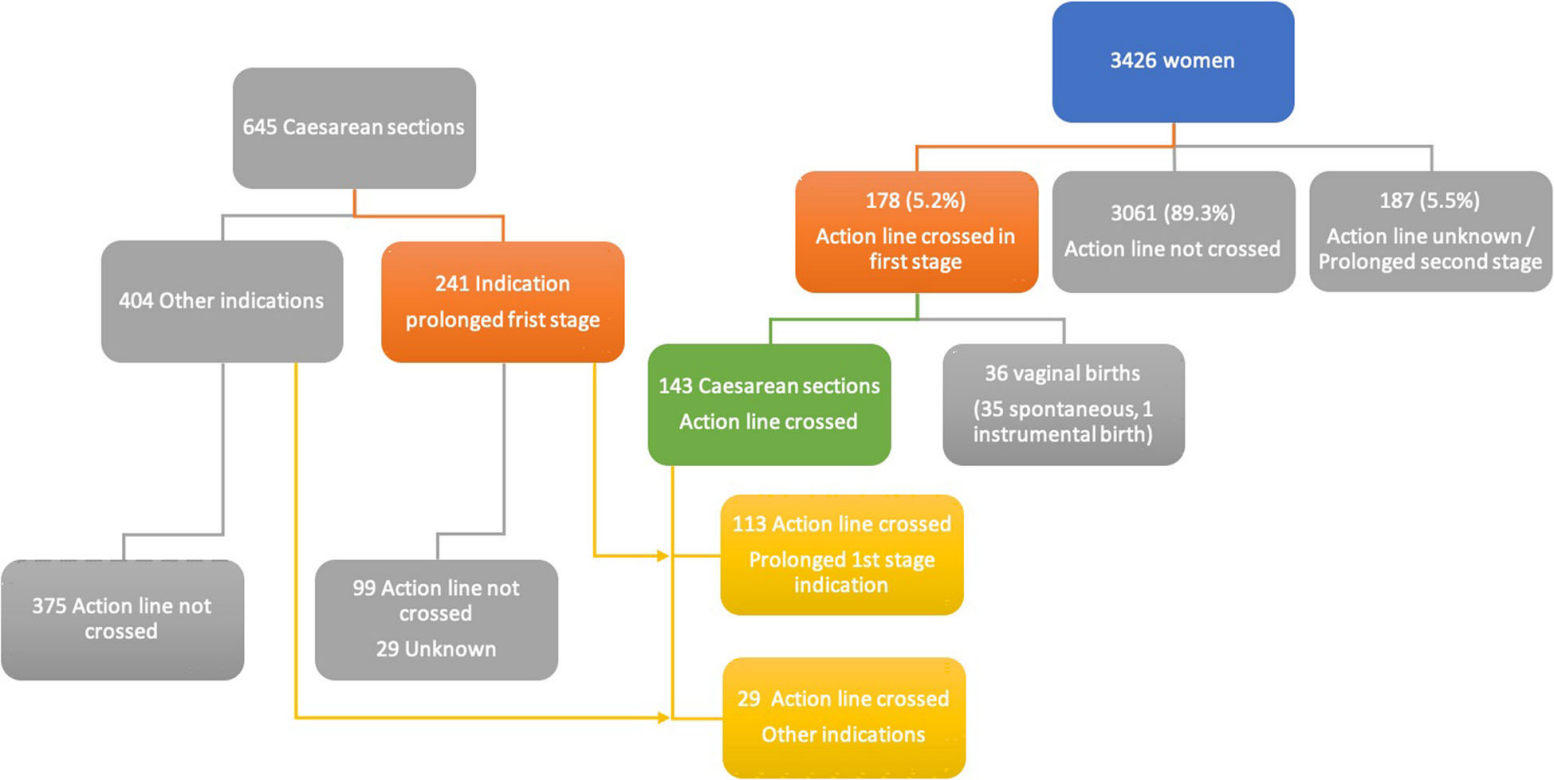
Flowchart of total number of women. In total 3426 women were included. On the right side of the flowchart the number of women who crossed the action line is highlighted (orange box, N = 178). The left side of the flowchart shows the total number of caesarean sections and the indications reflecting prolonged first stage (orange box, N = 241). The overlap between women with true prolonged labour (action line crossed) and prolonged first stage caesarean section is seen in the middle (yellow boxes)

In total, 178 women (5.2% of total) had crossed the action line in the first stage of labour (Fig. 2). Out of these 178, 142 (79,8%) gave birth by caesarean section, 35 (19,7%) by spontaneous vaginal birth and one had an assisted vaginal birth. Fifty-six (31.5%) women had spontaneously ruptured membranes and 28 (15.7%) had their membranes ruptured artificially. In 13 women (7.3%), membranes were still found to be intact during caesarean section and in 81 (45.5%) there was no documentation as to whether the membranes had ruptured. Augmentation with oxytocin was done in 21 women (11.8%) with prolonged first stage of labour. Ninety-three women were primi- and 85 multiparous. Among women without prolonged first stage of labour, amniotomy was performed in 385 women (12.6%) and for 65 (2.1%) labour was augmented with oxytocin infusion without crossing the action line. Table 1 compares characteristics of the 178 women with prolonged first stage of labour to women without prolonged labour (*N* = 3061, 89.3% of total) and Table 2 compares primi- and multiparous women from this total group of 3239 women, whereby 17 cases were excluded because of missing values on parity.

**Table 1:** Characteristics of women with prolonged first stage of labour compared to no prolonged labour (total *N* = 3239)

	Prolonged 1st stage of labour (action line crossed) ***N*** = 178	Not prolonged N = 3061
Age		
< 20	69 (38.8%)	747 (24.4%)
20–24	58 (32.6%)	883 (28.8%)
25–29	19 (10.7%)	575 (18.8%)
30–34	20 (11.2%)	431 (14.1%)
≥ 35	12 (6.7%)	333 (10.9%)
Unknown	0	92 (3.0%)
Gestational age		
< 32	0 (0.0%)	51 (1.7%)
32–34	4 (2.2%)	80 (2.6%)
35–36	34 (19.1%)	380 (12.4%)
37–39	61 (34.3%)	1109 (36.2%)
> 39	8 (4.5%)	117 (3.8%)
Unknown	71 (39.9%)	1324 (43.3%)
Parity		
0	95 (53.4%)	1055 (34.5%)
1	38 (21.3%)	659 (21.5%)
2	17 (9.6%)	503 (16.4%)
3	13 (7.3%)	374 (12.2%)
≥ 4	15 (8.4%)	453 (14.8%)
Unknown	0 (0.0%)	17 (0.6%)
Previous CS		
0	159 (89.3%)	2824 (92.3%)
1	19 (10.7%)	176 (5.7%)
≥ 2	0 (0.0%)	37 (1.2%)
Unknown	0 (0.0%)	24 (0.8%)
Type of pregnancy		
Singleton	171 (96.1%)	2995 (97.8%)
Twins	7 (3.9%)	66 (2.2%)
Birthweight		
< 1500	0 (0.0%)	29 (0.9%)
1500–1999	4 (2.2%)	58 (1.9%)
2000–2499	10 (5.6%)	321 (10.5%)
2500–2999	52 (29.2%)	1081 (35.3%)
3000–3499	67 (37.6%)	1191 (38.9%)
≥ 3500	37 (20.8%)	323 (10.6%)
Unknown	8 (4.5%)	58 (1.9%)
Mode of birth		
SVD	35 (19.7%)	2624 (85.7%)
AVD	1 (0.6%)	59 (1.9%)
CS	142 (79.8%)	378 (12.3%)
Oxytocin		
Yes	21 (11.8%)	65 (2.1%)
No	157 (88.2%)	2994 (97.8%)
Unknown	0 (0.0%)	2 (0.1%)
Rupture of membranes		
Spontaneous	56 (31.5%)	2377 (77.6%)
Artificial (amniotomy)	28 (15.7%)	385 (12.6%)
On CS	13 (7.3%)	69 (2.3%)
Unknown	81 (45.5%)	230 (7.5%)
	178	3061

*SVD* spontaneous vaginal delivery; *AVD* assisted vaginal delivery; *CS* caesarean section

**Table 2:** Primiparous and multiparous women compared (*N* = 3222, 17 women with unknown parity)

	Primiparous ***N*** = 1150	Multiparous ***N*** = 2072
Action line crossed in 1st stage	95 (8.3%)	83 (4.0%)
Action line not crossed	1055 (91.7%)	1989 (96.0%)
Amniotomy	164 (14.3%)	248 (12.0%)
Augmentation with oxytocin	54 (4.7%)	32 (1.5%)
SVD	905 (78.7%)	1742 (84.1%)
AVD	40 (3.5%)	20 (1.0%)
CS	205 (17.8%)	310 (15.0%)

*SVD* spontaneous vaginal delivery; *AVD* assisted vaginal delivery; *CS* caesarean section

### Caesarean sections

**Fig. 3:**
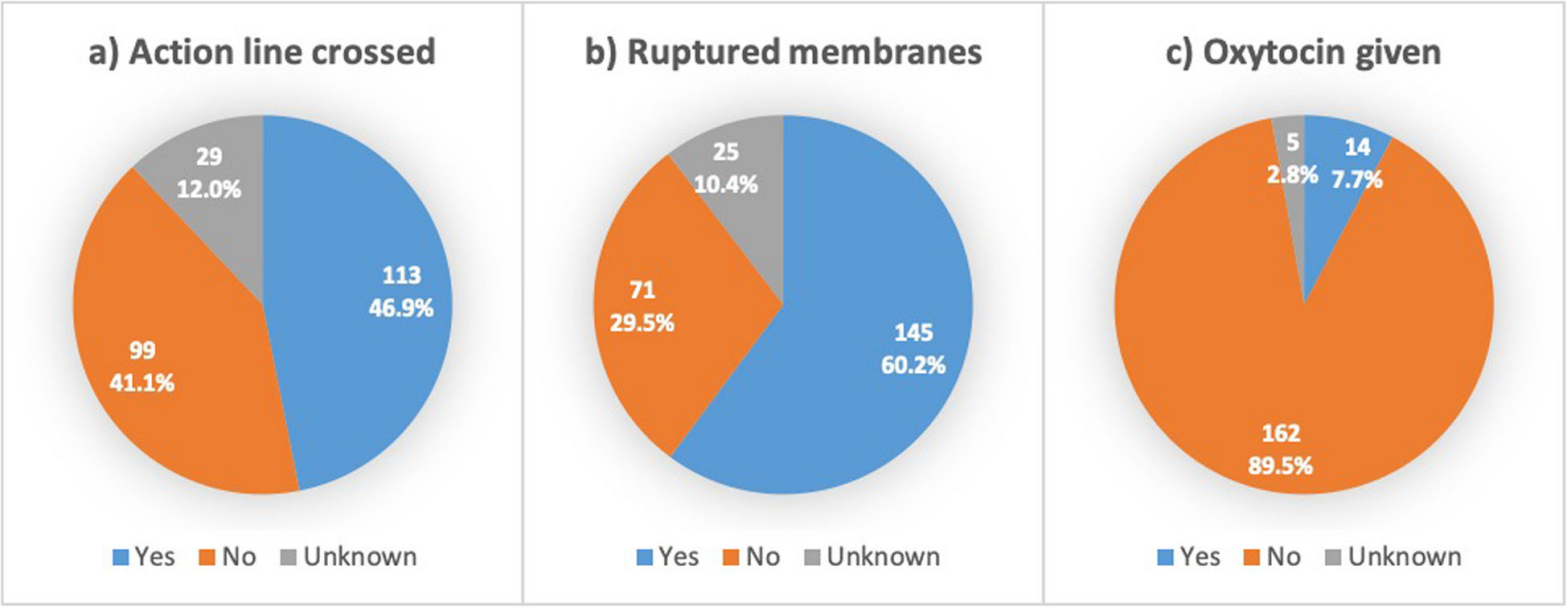
Interventions in caesareans for prolonged first stage of labour. a) Action line crossed (*N* = 241); b) Membranes ruptured at the start of caesarean section (N = 241); c) Augmentation of labour with oxytocin performed prior to caesarean section (*N* = 181, 60 women with previous caesarean section excluded)

Of all 3426 women, 645 women (18.8%) underwent caesarean section. Indications were grouped and displayed in Table 3. (Assumed) CPD was given as indication in 150 caesarean sections (23.3%) and in an additional 79 (12.2%) the indication was prolonged first stage of labour. In total 241 caesarean sections were performed with an indication similar to or reflecting prolonged first stage of labour (‘obstructed labour’, ‘CPD’, ‘prolonged first stage of labour’, ‘failed VBAC’, ‘failed induction’, ‘cervical dystocia’, ‘cervical oedema’ and ‘big fundus’, whereby 68 caesarean sections performed in the second stage were excluded). Out of these 241 women, 113 (46.9%) had cervical dilatation at the right side of the action line on the partograph, 99 (41.1%) did not cross the action line and in 29 (12.0%) it was unknown whether this had happened (Fig. 3). Seventy-one women (29.4%) still had intact membranes at the moment of caesarean section. Sixty of 241 women undergoing caesarean section for any indication reflecting prolonged first stage of labour had one previous caesarean section (24.9%), which can be considered a relative contra-indication for oxytocin augmentation in a setting without continuous monitoring [30]. Excluding these, only 14 out of 181 women (7.7%) without previous caesarean section, received oxytocin prior to surgery. It was noted that an additional 29 women gave birth by caesarean section with a different indication, but also had crossed the action line. These indications were fetal distress (*n* = 16), malpresentation (*n* = 5), prolonged second stage (*n* = 3), cord prolapse (n = 1), twins (n = 1), unknown (n = 3).

**Table 3:** Indications for caesarean sections

Indication	N (%)
Cephalopelvic disproportion	150 (23.3%)
Fetal distress	82 (12.7%)
Prolonged first stage of labour	79 (12.2%)
Two or more previous CS	57 (8.8%)
Fetal malpresentation (e.g. breech)	53 (8.2%)
Prolonged second stage of labour	30 (4.7%)
Failed VBAC	20 (3.1%)
APH / placental abruption	19 (3.0%)
Obstructed labour	16 (2.5%)
Eclampsia	14 (2.2%)
Pre-eclampsia	10 (1.6%)
Cervical dystocia	10 (1.6%)
Cord prolapse	9 (1.4%)
Other	42 (7.0%)
Missing	54 (8.4%)
Total	645 (100%)

*CS* caesarean section; *VBAC* vaginal birth after caesarean; *APH* antepartum haemorrhage

### Neonatal outcomes with oxytocin

There was no significant difference in the proportion of low one-minute Apgar (< 7) scores of neonates from women who had their labour augmented (9.3%) versus those who did not (7.5%; OR 1.26; 95% confidence interval (CI) 0.60–2.64). For five-minute Apgar scores < 7 it was 3.5% versus 2.3% (OR 1.55; 95% CI 0.48–5.04).

## Discussion

True prolonged first stage of labour with crossing of the action line occurred only in 5.5% of births in our setting and was therefore not very frequent. In case of prolonged first stage of labour, caesarean section was commonly the performed intervention. Interventions like amniotomy and oxytocin augmentation appear to be underused, often in situations with clear indications and no contra-indications. Although documentation on rupture of membranes was suboptimal, we found a significant number of caesarean sections for prolonged labour being performed with intact membranes (29.4%). Despite the fact that local and national protocols promote active support of labour, it does not seem to be standard practice [30]. Many women without clear evidence of prolonged labour on the partograph still had their labour augmented with oxytocin. Possible reasons for such deviations from what should be standard clinical care could be insufficient training or skills, or fear of complications of obstetric interventions. Additional quantitative and qualitative studies may provide insight into these issues.

As expected, prolonged first stage of labour is more common in primiparous women and for this group it is of even greater importance to utilize methods to augment labour. Preventing the first caesarean section is crucial. It will have tremendously positive impact on a woman’s subsequent fertile life, reducing chances of future complications and additional caesarean sections. This is particularly important in a country with high fertility rates such as Malawi. While the overall use of oxytocin was low, it was clearly more utilized in primiparous compared to multiparous women.

Although it is easy to state that protocols need to be followed better based on our results, optimal solutions to prevent unnecessary caesarean sections in the first stage of labour are most likely more challenging. As noted by Betran et al. [3], overuse of caesarean sections is caused by many factors and seems challenging to be reduced with simple measures. We deem it important to acknowledge that our results do not reflect capabilities of individual health workers, but could indicate a more systemic issue. In a setting like Malawi, scarcity of resources sometimes impairs quality of care, although it appears that oxytocin, amniotomy hooks and intravenous cannulas have been present in the facility and Malawi at large [33]. Furthermore, preventing caesarean section with active support of labour saves surgical resources which might be needed for emergency procedures [34].

A review of management of prolonged labour in Rwandan hospitals, a setting with similarities to the Malawian setting, showed comparable results [35]. The authors found unnecessary use of oxytocin augmentation in women without prolonged labour and identified many women who had given birth by caesarean section with intact membranes. However, among women who crossed the action line a large majority had received oxytocin, which contrasts with our findings.

A diagnosis of prolonged first stage of labour under the assumption of CPD is made rather quickly and frequently in our setting without signs of prolonged labour on the partograph. Suboptimal monitoring may contribute to early caesarean sections, which might be considered a safe choice at that stage of labour. Difficulties in monitoring labour progress are described in different settings, including Tanzania and Ethiopia [36, 37]. Maternal and fetal monitoring is a crucial aspect of labour process and with proper monitoring of labour and fetal condition, prolonged labour can often be managed adequately using non-invasive interventions.

A limitation of our study is that outcomes depend on the quality of case files and partographs. While clearly incomplete partographs were excluded, analysed partographs may still lack important data, for example the exact moment of amniotomy or the use and duration of oxytocin augmentation. Whilst hospital and national protocols require documentation of these details in the designated areas of the partograph, it is expected that there are situations in which this did not happen, as was previously documented in similar settings [20]. There has been a large number of most likely randomly distributed missing values, of which gestational age and labour progress are among the most important. We have taken effort to report these missing values for the different variables of importance. There is a chance of selection bias since women with incomplete and missing partographs were excluded, but with the partograph holding the most important information on pregnancy and labour, medical record analysis without a well-filled partograph is almost impossible.

## Conclusion

The diagnosis prolonged first stage of labour was often made without being evident from labour tracings and true prolonged labour was uncommon. Caesarean section was frequently performed on the suspicion of prolonged labour. Amniotomy and controlled augmentation with oxytocin might be underused in some low-resource settings and further studies are needed to explore barriers for active support of labour in order to make efforts to reduce unnecessary caesarean sections.

## Supplementary Information

**Additional file 1.**

## Data Availability

The datasets used and/or analysed during the current study are available from the corresponding author on reasonable request.

## Abbreviations

CPD: Cephalopelvic disproportion
WHO: World Health Organization
HMIS: Health Management Information System
SVD: Spontaneous vaginal delivery
AVD: Assisted vaginal delivery
VBAC: Vaginal birth after caesarean
APH: Antepartum haemorrhage
